# Bromodomain and extraterminal inhibitors block the Epstein-Barr virus lytic cycle at two distinct steps

**DOI:** 10.1074/jbc.M116.751644

**Published:** 2017-06-06

**Authors:** Kristin M. Keck, Stephanie A. Moquin, Amanda He, Samantha G. Fernandez, Jessica J. Somberg, Stephanie M. Liu, Delsy M. Martinez, JJ L. Miranda

**Affiliations:** From the § Department of Cellular and Molecular Pharmacology, University of California, San Francisco (UCSF), California 94158 and; the ‡ Gladstone Institute of Virology and Immunology, San Francisco, California 94158

**Keywords:** bromodomain-containing protein 4 (BRD4), chromatin, DNA replication, herpesvirus, transcription, BET inhibitors, Epstein-Barr virus, JQ1

## Abstract

Lytic infection by the Epstein-Barr virus (EBV) poses numerous health risks, such as infectious mononucleosis and lymphoproliferative disorder. Proteins in the bromodomain and extraterminal (BET) family regulate multiple stages of viral life cycles and provide promising intervention targets. Synthetic small molecules can bind to the bromodomains and disrupt function by preventing recognition of acetylated lysine substrates. We demonstrate that JQ1 and other BET inhibitors block two different steps in the sequential cascade of the EBV lytic cycle. BET inhibitors prevent expression of the viral immediate-early protein BZLF1. JQ1 alters transcription of genes controlled by the host protein BACH1, and BACH1 knockdown reduces BZLF1 expression. BET proteins also localize to the lytic origin of replication (OriLyt) genetic elements, and BET inhibitors prevent viral late gene expression. There JQ1 reduces BRD4 recruitment during reactivation to preclude replication initiation. This represents a rarely observed dual mode of action for drugs.

## Introduction

Modern antiviral drugs generally share two characteristics: the target is a single protein, and that target acts at one distinct step in the viral life cycle (1). Acyclovir treats herpes by inhibiting the DNA polymerase. Azidothymidine treats HIV by inhibiting the reverse transcriptase. Sofosbuvir treats hepatitis C by inhibiting the RNA polymerase. Despite tremendous success, single-target/single-mechanism antiviral drugs have key weaknesses. Acquisition of viral resistance occurs frequently. A single target provides only a single point of intervention and potency. How then can we in the field improve upon the current paradigm of drug design? We take our cues from the success of two efforts: combination antiretroviral therapy and polypharmacology. HIV medication is now often delivered as a combination of single-target drugs (2). Chemical biologists are also challenging the single-target/single-mechanism paradigm by systematically optimizing the broad spectrum of targets hit by a drug (3). Off-target effects are not avoided but rather specifically chosen to generate additive or synergistic interactions with other known targets. Here we report the serendipitous discovery of polypharmacological activity by a small molecule epigenetic regulator that acts against the Epstein-Barr virus (EBV).^5^

**Figure 1.**
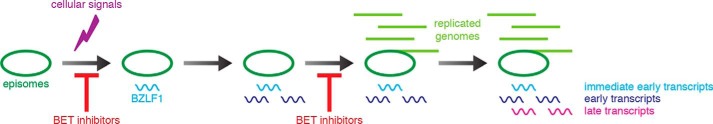
**Schematic of the EBV lytic cycle and BET inhibitor points of intervention.** Each *arrow* indicates one sequential step in the cascade: cellular signals induce immediate-early gene expression, immediate-early proteins transactivate early genes, early gene products license lytic DNA replication, and lytic DNA replication promotes late gene expression. Data presented in this study provide evidence that BET inhibitors suppress immediate-early gene expression and lytic DNA replication.

EBV maintains a lifelong infection in over 90% of adults worldwide. Like other herpesviruses, EBV infection cycles between latent (4) and lytic (5) forms. During the latent phase, the ∼170-kb genome is maintained as an episome, and transcription is limited to a dozen or fewer latent genes. The lytic cycle involves expression of ∼100 genes and leads to production of viral progeny through a sequential cascade. This stepwise process begins upon either initial infection or reactivation from latency (summarized in Fig. 1). First, cellular signals lead to expression of immediate-early genes, among which *BZLF1* is necessary and sufficient to promote downstream events (6). The encoded proteins drive expression of early genes whose products allow for replication of the viral genome and finally expression of late genes. Although latent infection is implicated in the development of many cancers, such as Burkitt lymphoma and nasopharyngeal carcinoma (4), lytic infection causes infectious mononucleosis (7) and drives post-transplantation lymphoproliferative disorder (8).

Proteins in the bromodomain and extraterminal (BET) family regulate multiple stages of viral life cycles. The bovine papilloma virus protein E2 binds the human protein BRD4 directly and colocalizes on mitotic chromosomes to attach viruses for proper segregation (9, 10). Mutations in E2 that perturb BRD4 binding abrogate attachment. Similar observations have been made with the Kaposi sarcoma-associated herpesvirus protein latency-associated nuclear antigen and BRD4 (11). BRD4 also activates EBV enhancer (12) and promoter (13) function to modulate gene expression. In addition to promoting viral propagation, BET proteins can also inhibit production. BRD2 and BRD4 suppress reactivation of latent HIV by antagonizing transcription elongation (14–17).

JQ1 (18) and I-BET (19) are inhibitors of BET protein bromodomains that demonstrate strong affinity for the three family members widely expressed in human tissues: BRD2, BRD3, and BRD4. Competitive binding to the two tandem bromodomains prevents recognition of acetylated lysine substrates. Although JQ1 targets both bromodomains with similar affinity (18), the compound RVX-208 preferentially binds to the second bromodomain (20). Given multiple host protein targets and multiple functions in viral life cycles, JQ1 and other BET inhibitors present intriguing potential for polypharmacological inhibition of viral replication. We tested this hypothesis with EBV and discovered two different points of intervention.

## Results

### BET inhibitors block immediate-early transcription

**Figure 2.**
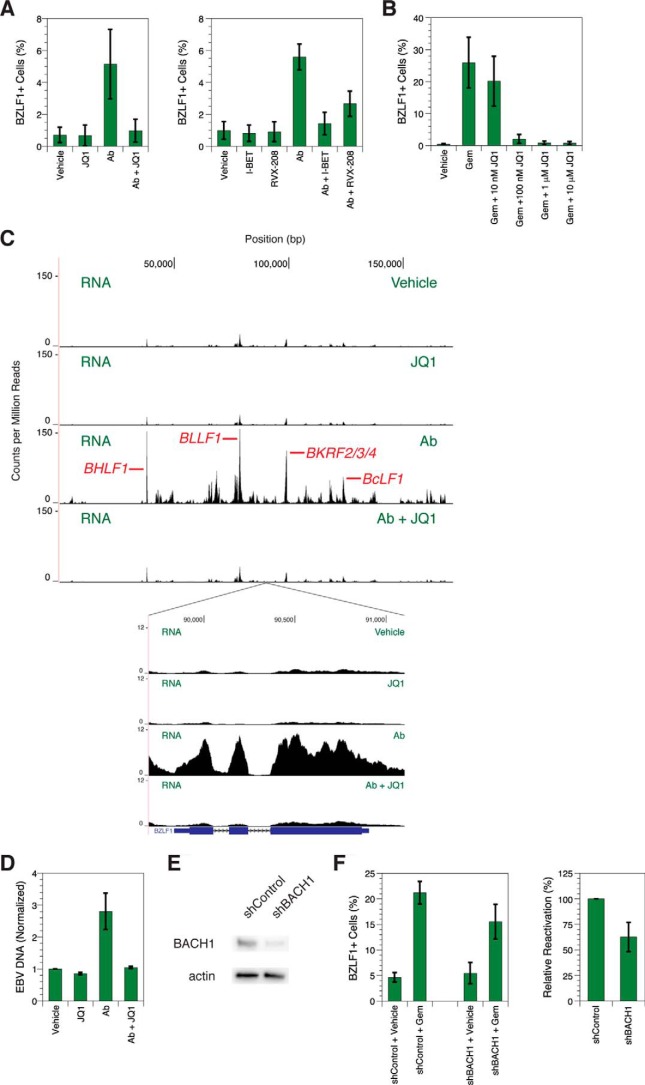
**BET inhibitors suppress *BZLF1* expression.** *A*, flow cytometry analysis of BZLF1 staining in MutuI cells treated with antibody (*Ab*). *Error bars* show the standard deviation of *n* = 8 (*left*) or *n* = 4 (*right*) replicates. *B*, flow cytometry of BZLF1 staining in MutuI cells treated with gemcitabine (*Gem*). *Error bars* show the standard deviation of *n* = 4 replicates. *C*, RNA-seq profiles of treated MutuI cells showing the entire EBV genome (*top*) or the region containing the *BZLF1* gene (*inset*). *Axes* denote genomic position in base pairs and counts per million mapped reads. Some major peaks corresponding to lytic gene expression are labeled. *Below* the *inset*, the *BZLF1* gene is shown in schematic form where *blocks* represent exons and *lines* with *arrows* represent introns. Results are representative of three independent biological replicates. *D*, -fold change in EBV DNA from treated MutuI cells based on deep sequencing of chromatin. EBV content was calculated as a percentage of total sequenced DNA, and for each set, EBV DNA percentage was normalized to that in the vehicle-treated sample. *Error bars* represent the standard deviation of *n* = 3 replicates. *E*, Western blots of BACH1 expression levels in MutuI cells after shRNA-mediated knockdown. β-Actin expression levels are shown as normalization controls. *F*, flow cytometry of BZLF1 staining in MutuI cells treated with gemcitabine during BACH1 knockdown. Relative reactivation is calculated as the increase in BZLF1-positive cell percentage relative to the control. *Error bars* show the standard deviation of *n* = 5 replicates.

Here we present evidence that BET inhibitors block the EBV lytic cycle at two distinct steps, the first occurring before immediate-early transcription. We measured expression of BZLF1, the immediate-early transactivator that serves as a marker for the lytic cycle, using flow cytometry. With MutuI, an EBV-positive Burkitt lymphoma line, only ∼1% of cells display background spontaneous reactivation (Fig. 2*A*). Cells treated with 1 μm JQ1, I-BET, or RVX-208 alone similarly yield a low percentage of cells positive for BZLF1. Antibodies raised against human immunoglobulin cross-link the B cell receptor and reactivate EBV from latency (21). For cells grown in the presence of antibody, pretreatment with BET inhibitors instead of vehicle decreases the percentage of BZLF1-positive cells, which indicates fewer cells containing lytic EBV. JQ1 and I-BET both reduce antibody-induced BZLF1 expression to approximately the level seen without antibody treatment. Used at the same concentration as JQ1, RVX-208 results in slightly less inhibition, implicating bromodomain 1 of the BET proteins in initiation of the EBV lytic cycle. To measure dose-dependent inhibition by JQ1, we treated cells with the cytotoxic chemotherapy drug gemcitabine, which also induces lytic progression (22). We measured an IC_50_ of 20 ± 9 nm (Fig. 2*B*), a concentration consistent with the affinity of the small molecule for BET bromodomains (18). We therefore chose to perform future experiments at a BET inhibitor concentration of 1 μm, the approximate lowest dose that yields maximum efficacy.

JQ1 blocking BZLF1 protein production should also prevent downstream transcription of lytic genes and replication of viral DNA. To confirm this inhibition of transcription, we performed RNA-seq on cells induced by antibody with or without 1 μm JQ1 pretreatment (Fig. 2*C*). We controlled for pleiotropic toxicity by verifying that JQ1-treated cells show similar growth, 97 ± 12%, and viability, 102 ± 1%, compared with untreated cells. Vehicle-treated lines display a transcription profile consisting predominantly of low-abundance lytic gene signals from background spontaneous reactivation (23, 24). Cells grown in the presence of antibody show increased transcription throughout the EBV genome similar to induction observed previously (24). The highest peaks correspond to lytic genes, some of which are labeled in Fig. 2*C*. In contrast, cells pretreated with JQ1 before antibody exposure yield an overall profile similar to that from cells not treated with antibody, confirming that JQ1-mediated inhibition of BZLF1 expression also prevents increased transcription of downstream lytic genes. Similar to the rest of the transcriptome, *BZLF1* expression only increases above background levels in cells incubated with antibody alone (Fig. 2*C*, *inset*). Thus, we know that the lack of BZLF1 protein induction detected by flow cytometry after JQ1 treatment (Fig. 2*A*) is due to lack of *BZLF1* gene expression rather than post-transcriptional regulation. We also confirmed that replication of viral DNA is inhibited upon JQ1 pretreatment by deep sequencing all DNA extracted from cells and calculating the increase in viral genomes. The pattern of the results is similar to that seen with lytic gene transcription: viral DNA increases with antibody exposure but decreases back to background levels with JQ1 pretreatment prior to antibody exposure (Fig. 2*D*). Thus, JQ1 completely suppresses the EBV lytic cycle even at downstream readouts, speaking to the strong efficacy of inhibition.

### BET proteins localize to the lytic origins of replication

**Figure 3.**
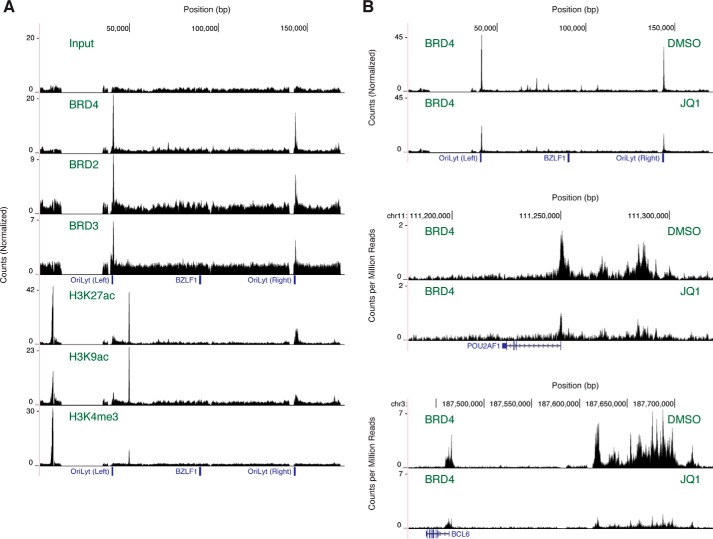
**BET proteins bind the lytic origins of replication.** *A*, ChIP-seq mapping of BET protein and histone modification occupancy on the EBV genome in untreated MutuI cells. Input DNA (*top*) serves as a control reference. Results are representative of two independent biological replicates. *B*, ChIP-seq mapping of BRD4 occupancy on the EBV and human genomes in MutuI cells treated with JQ1. Results are representative of two independent biological replicates. For the EBV genome, locations of the *BZLF1* gene and the left and right OriLyt elements are indicated. Occupancy is calculated as enrichment over the background baseline. For the human genome, the *POU2AF1* and *BCL6* genes are shown in schematic form where *blocks* represent exons and *lines* with *arrows* represent introns. Occupancy is calculated as counts per million mapped reads.

Our first hint that BET inhibitors act at multiple steps in the viral life cycle came when we discovered that BET proteins bind the EBV genome at the two lytic origin of replication (OriLyt) elements. Given that BET inhibitors prevent *BZLF1* expression, we suspected that BET proteins would localize to the *BZLF1* promoter. To test this possibility and to simultaneously check for potential binding elsewhere, we probed the entire EBV genome for BRD2, BRD3, and BRD4 occupancy with ChIP-seq (Fig. 3*A*). Contrary to our suspicion, we did not detect noticeable enrichment in the region near the *BZLF1* transcription start site at ∼90 kb on the EBV genome. Much to our surprise, however, we detected strong signals of occupancy for all three BET proteins at ∼41 and ∼144 kb within the 3′ edge of the two OriLyt elements (25) genetically defined as nucleotides 40301–41293 and 143207–144444. Histone modifications that colocalize with BET proteins in these regions (Fig. 3*A*) are also consistent with bromodomain function. Acetylated H3K9 and H3K27, which may serve as bromodomain-binding partners, are enriched at these sites. The BET protein-bound regions lack peaks of H3K4 trimethylation, suggesting the assembly of enhancer-like as opposed to promoter-like chromatin (26).

We also measured the effect of JQ1 on BRD4 occupancy at the OriLyt elements. Although our initial ChIP-seq protocol readily detected BRD4 occupancy on the high-copy number EBV episome, we could not readily identify occupancy on the human genome. To improve sensitivity so that we could compare binding in response to BET inhibitor treatment between sites on both genomes, we increased the number of cells processed and repeated the ChIP-seq experiment. Among many other sites, BRD4 has been shown to bind superenhancers of the *POUF2AF1* and *BCL6* genes in B cell lymphoma lines (27). In our own experiments, as expected, 1 μm JQ1 substantially reduces BRD4 occupancy both at these superenhancers and at associated promoter-proximal regions (Fig. 3*B*). JQ1 also reduces BRD4 binding to the viral OriLyt elements ∼2-fold. Although this reduction of occupancy in the EBV genome is weaker than effects observed in the human genome, similar behavior at the viral OriLyt elements further validates our identification of this enriched occupancy as BET protein-binding events.

If BET proteins do not directly bind the *BZLF1* promoter to drive expression, then we surmise that BET inhibitor activity at that site occurs through an indirect mechanism involving other host proteins. Our RNA-seq data identified ∼3000 differentially regulated human genes in response to 1 μm JQ1 treatment. Pathway analysis reveals 10 enriched groups of genes either known or predicted to be controlled by the transcription factors FOXP3, STAT6, SPI1, KLF1, E2F4, EBF1, BACH1, YY1, NFE2L2, and TAL1. We followed up on these leads by depleting individual proteins and measuring the effect on viral reactivation. Lentivirus shRNA reduces BACH1 expression by ∼70% compared with a control non-targeting shRNA (Fig. 2*E*). This BACH1 knockdown reduces the increase in BZLF1 expression induced by gemcitabine ∼2-fold (Fig. 2*F*). Other candidate factors may also mediate the effect of BET inhibitors on viral transcription, but our preliminary results suggest that JQ1 perturbs BACH1 function to decrease immediate-early protein production.

### BET inhibitors prevent lytic DNA replication

**Figure 4.**
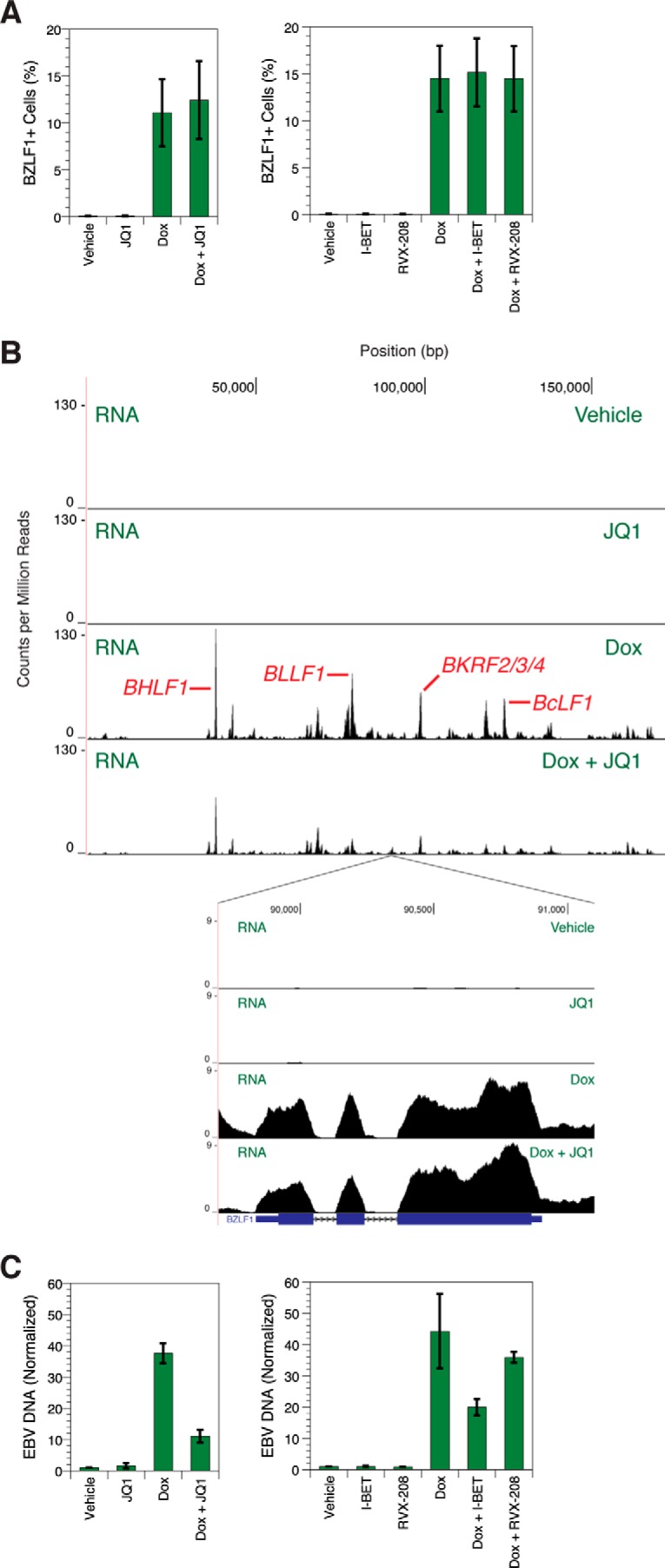
**BET inhibitors suppress lytic DNA replication.** *A*, flow cytometry analysis of BZLF1 staining in Akata-Zta cells treated with doxycycline (*Dox*). *Error bars* show the standard deviation of *n* = 4 replicates. *B*, RNA-seq profiles of treated Akata-Zta cells showing the entire EBV genome (*top*) or the region containing the *BZLF1* gene (*inset*). *Axes* denote genomic position in base pairs and counts per million mapped reads. Some major peaks corresponding to lytic gene expression are labeled. *Below* the *inset*, the *BZLF1* gene is shown in schematic form where *blocks* represent exons and *lines* with *arrows* represent introns. Results are representative of three independent biological replicates. *C*, -fold change in EBV DNA from treated Akata-Zta cells based on deep sequencing of chromatin. EBV content was calculated as a percentage of total sequenced DNA, and for each set, EBV DNA was normalized to that in the vehicle-treated sample. *Error bars* represent the standard deviation of *n* = 3 replicates.

To demonstrate that BET inhibitors act at a second step in the viral life cycle, we determined that JQ1 blocks EBV DNA replication despite ectopic *BZLF1* expression. Because BRD2, BRD3, and BRD4 bind the lytic origins of replication, we hypothesized that BET inhibitors could perturb the function of that genetic element. Prevention of *BZLF1* expression in MutuI cells by JQ1 precludes testing for this effect because blocking immediate-early transcription abrogates all downstream events, including lytic DNA replication (Fig. 2). To perform a classical epistasis experiment and determine whether JQ1 treatment regulates viral transcription downstream of *BZLF1* expression, we studied Akata-Zta (28), an EBV-positive cell line with a doxycycline-inducible and plasmid-borne *BZLF1* gene. If the effect of BET protein inhibition by JQ1 were only upstream of *BZLF1* transcription, then ectopic expression of *BZLF1* should cause reactivation of EBV even in the presence of JQ1. We first ensured that BET inhibitors did not reduce ectopic *BZLF1* expression by verifying that pretreatment before addition of doxycycline did not affect the percentage of BZLF1-positive cells (Fig. 4*A*). RNA-seq verified that, as expected, incubation with doxycycline and consequent lytic cycle induction cause transcription to increase throughout the EBV genome (Fig. 4*B*).

If DNA replication were perturbed by JQ1, the sequential ordering of EBV transcription predicts that late gene expression would be reduced without affecting early gene expression. When Akata-Zta cells are pretreated with 1 μm JQ1 before exposure to doxycycline, total transcription of *BZLF1* does not change (Fig. 4*B*, *inset*), whereas expression is reduced at many other genes (Fig. 4*B*). Again, we controlled for pleiotropic toxicity by verifying that JQ1-treated cells showed similar growth, 79 ± 23%, and viability, 101 ± 1%, compared with untreated cells. The overlapping nature of EBV gene organization often confounds analysis, so we measured expression only at non-overlapping RNA segments. We found that, although none of 18 early genes are perturbed by JQ1 pretreatment, 12 of 18 late genes show significantly decreased expression (Table 1). RNA levels for 11 of the 12 differentially regulated genes change by greater than 2-fold. Similar but less pronounced effects are observed with I-BET pretreatment. None of 18 early genes and four of 18 late genes show significantly decreased expression (Table 2), but the RNA level change was less than 2-fold. We also verified that viral transcripts needed for OriLyt function were not disturbed. In addition to *BZLF1*, eight genes are necessary for lytic DNA replication (29). Of these eight, overlapping transcripts precluded unambiguous measurement of *BMRF1* and *BMLF1* expression. Levels of the six other genes, *BALF5*, *BALF2*, *BSLF1*, *BBLF4*, *BBLF2/BBLF3*, and *BRLF1*, however, do not decrease upon treatment with either JQ1 or I-BET (Tables 1 and 2). Down-regulation of expression specific to late genes even in the presence of the transcripts required for OriLyt function points to a blockade of lytic DNA replication.

**Table 1:** **EBV genes differentially regulated by JQ1 during viral reactivation** Bold font indicates differentially regulated genes with a *p* value <0.05.

Gene	Coordinates	Log_2_ -fold change	*p* value
**Late (12 of 18 change expression level)**			
***BNRF1***	**1691–5407**	**−1.50**	**0.010**
***BCRF1***	**9631–10262**	**−0.88**	**0.004**
*BOLF1*	59905–62728	+0.12	0.837
*BORF1*	63035–63880	+0.07	0.908
*BSRF1*	74594–75316	−0.02	0.739
***BLRF1***	**76232–76574**	**−1.41**	**0.031**
***BLLF1***	**77764–79904**	**−1.79**	**0.001**
***BZLF2***	**89483–89828**	**−1.90**	**0.004**
***BRRF2***	**93955–95631**	**−1.44**	**0.009**
***BKRF2***	**97655–98064**	**−1.71**	**0.015**
*BBRF1*	101972–103659	−0.70	0.064
***BBRF3***	**106751–108075**	**−1.37**	**0.032**
***BGRF1*/*BDRF1***	**112826–113190, 117017–118064**	**−1.08**	**0.028**
***BDLF1***	**120189–121018**	**−1.52**	**0.003**
***BcLF1***	**121099–125072**	**−1.79**	**0.004**
*BXRF1*	132847–133012	−0.73	0.098
***BVRF2***	**135628–136330**	**−1.42**	**0.010**
*BILF2*	137464–138282	−0.74	0.080

**Early (0 of 18 change expression level)**			
*BHLF1*	38014–40529	−0.92	0.069
*BHRF1*	41471–43251	−1.14	0.129
*BFLF1*	44794–46235	−0.68	0.139
*BaRF1*	66601–67551	−0.31	0.377
*BSLF1**^a^*	72069–74593	−0.48	0.208
*BLLF3*	75320–76218	−0.31	0.330
*BRLF1**^a^*	90907–92727	−0.03	0.713
*BRRF1*	92898–93827	−0.35	0.255
*BBLF4**^a^*	99537–101587	−0.35	0.258
*BBLF2*/*BBLF*3*^a^*	104503–105098, 105227–106692	+0.03	0.793
*BcRF1*	125423–127415	−0.39	0.324
*BXLF1*	131022–132570	+0.11	0.859
*LF3*	140692–143711	−0.25	0.306
*BALF5**^a^*	152642–155265	−0.28	0.214
*BALF3*	160532–160549	−0.47	0.209
*BALF2**^a^*	160909–164356	−0.42	0.269
*BALF1*	164388–164984	−0.82	0.239
*BARF1*	165008–165712	−0.71	0.166

**Unassigned (0 of 6 change expression level)**			
*BCLT1*	5868–6136	−0.66	0.157
*BCLT2*	6172–6475	−0.51	0.216
*BFRF1A*	46281–46543	−0.49	0.158
*BGLF3*	111830–112649	−0.95	0.079
*BDLF3.5*	116767–116926	−0.86	0.064
*BVLF1*	134887–135431	−0.44	0.332

*^a^* Protein product required for lytic DNA replication.

**Table 2:** **EBV genes differentially regulated by I-BET during viral reactivation** Bold font indicates differentially regulated genes with a *p* value <0.05.

Gene	Coordinates	Log_2_ -fold change	*p* value
**Late (4 of 18 change expression level)**			
***BNRF1***	**1691–5407**	**−0.68**	**0.038**
*BCRF1*	9631–10262	+0.03	0.929
*BOLF1*	59905–62728	−0.29	0.485
*BORF1*	63035–63880	−0.22	0.621
*BSRF1*	74594–75316	+0.27	0.418
*BLRF1*	76232–76574	−0.54	0.127
*BLLF1*	77764–79904	−0.73	0.112
*BZLF2*	89483–89828	−0.94	0.233
*BRRF2*	93955–95631	−0.54	0.115
***BKRF2***	**97655–98064**	**−0.78**	**0.009**
*BBRF1*	101972–103659	−0.11	0.504
*BBRF3*	106751–108075	−0.48	0.088
***BGRF1*/*BDRF1***	**112826–113190, 117017–118064**	**−0.43**	**0.008**
*BDLF1*	120189–121018	−0.60	0.333
*BcLF1*	121099–125072	−0.80	0.075
*BXRF1*	132847–133012	−0.14	0.611
*BVRF2*	135628–136330	−0.65	0.095
***BILF2***	**137464–138282**	**−0.28**	**0.017**

**Early (0 of 18 change expression level)**			
*BHLF1*	38014–40529	−0.45	0.297
*BHRF1*	41471–43251	−0.56	0.054
*BFLF1*	44794–46235	−0.28	0.473
*BaRF1*	66601–67551	−0.23	0.445
*BSLF1**^a^*	72069–74593	−0.20	0.578
*BLLF3*	75320–76218	−0.21	0.458
*BRLF1**^a^*	90907–92727	+0.07	0.796
*BRRF1*	92898–93827	−0.10	0.689
*BBLF4**^a^*	99537–101587	−0.19	0.180
*BBLF2*/*BBLF3**^a^*	104503–105098, 105227–106692	+0.10	0.653
*BcRF1*	125423–127415	+0.04	0.801
*BXLF1*	131022–132570	+0.22	0.618
*LF3*	140692–143711	−0.10	0.667
*BALF5**^a^*	152642–155265	−0.22	0.311
*BALF3*	160532–160549	−0.59	0.216
*BALF2**^a^*	160909–164356	−0.38	0.375
*BALF1*	164388–164984	−0.24	0.356
BARF1	165008–165712	−0.38	0.078

**Unassigned (0 of 6 change expression level)**			
*BCLT1*	5868–6136	+0.26	0.995
*BCLT2*	6172–6475	+0.18	0.827
*BFRF1A*	46281–46543	−0.13	0.794
*BGLF3*	111830–112649	−0.30	0.348
*BDLF3.5*	116767–116926	−0.11	0.764
*BVLF1*	134887–135431	−0.22	0.512

*^a^* Protein product required for lytic DNA replication.

To directly verify a replication defect, we measured the viral DNA content of cells. We found that 1 μm JQ1 pretreatment reduced the percentage of EBV DNA in cells by ∼3-fold compared with doxycycline treatment alone (Fig. 4*C*). I-BET pretreatment reduced EBV DNA content by ∼2-fold, and this weaker inhibition of DNA replication relative to that by JQ1 is consistent with the smaller RNA-seq perturbations. RVX-208 yields no reduction, again emphasizing the primary role of bromodomain 1 in inhibition of the EBV life cycle.

**Figure 5.**
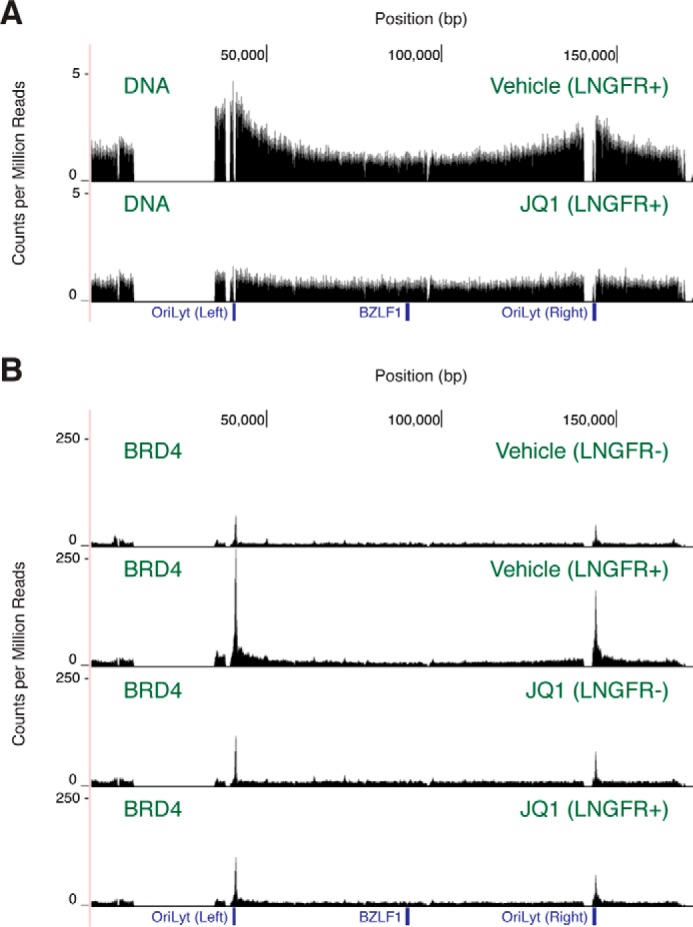
**JQ1 prevents BRD4 recruitment to the lytic origins of replication.** *A*, EBV DNA content based on deep sequencing of chromatin from Akata-Zta cells treated with JQ1. Results are representative of two independent biological replicates. *B*, ChIP-seq mapping of BRD4 occupancy on the EBV genome in Akata-Zta cells treated with JQ1. Results are representative of two independent biological replicates. All conditions include acyclovir pretreatment and reactivation with doxycycline. Cells containing lytic EBV were purified based on positive LNGFR expression. For the EBV genome, locations of the *BZLF1* gene and the left and right OriLyt elements are indicated. Occupancy is calculated as counts per million mapped reads.

We further probed the step at which lytic replication is blocked by measuring the appearance of newly synthesized portions in preparations of bulk DNA. Akata-Zta cells contain the low-affinity nerve growth factor receptor (*LNGFR*) gene under control of the same bidirectional tetracycline-inducible promoter that expresses the BZLF1 protein. Magnetic purification of this cell surface marker allows for separation of cells containing latent and reactivated EBV. When Akata-Zta cells are pretreated with acyclovir before exposure to doxycycline, we detect more DNA content near both OriLyt elements in cells containing lytic episomes (Fig. 5*A*). The signal arises from abortive DNA replication that is initiated prior to incorporation of acyclovir and consequent chain termination during elongation. When 1 μm JQ1 pretreatment is added, this abortive replication is not detected. Thus, JQ1 blocks the initiation of lytic DNA replication.

Although JQ1 reduces BRD4 binding to the OriLyt elements during latency (Fig. 3*B*), we suspected that larger perturbations may occur during reactivation. We therefore measured differences in BRD4 occupancy between latent and lytic episomes with ChIP-seq (Fig. 5*B*). Akata-Zta cells were reactivated with doxycycline but also pretreated with acyclovir to prevent lytic synthesis of linear genomes that may confound measurement of protein binding to circular episomes. Populations containing latent and lytic EBV were separated based on LNGFR expression. In the absence of JQ1, BRD4 occupancy increases ∼4–12-fold at the lytic origins of replication upon reactivation. In the presence of JQ1, occupancy increases only ∼1–2-fold. JQ1 reduces the change in enrichment by ∼4–5-fold. This prevention of BRD4 recruitment may underlie the defect in lytic DNA replication initiation.

## Discussion

BET inhibitors display specific activity against EBV. Dual-action transcriptional inhibition does not appear to be the result of a nonspecific antiviral host response against episomal DNA viruses. JQ1 reduces replication of the John Cunningham polyomavirus through inhibition of BRD4-mediated NF-κB coactivation (30). Opposite effects are observed with the herpes simplex virus as BET inhibitors promote lytic infection (31) and stimulate reactivation from latency (32) by enhancing levels of positive transcription elongation factor b on viral promoters. Results with the Kaposi sarcoma-associated herpesvirus depend on context. JQ1 does not induce lytic replication usually observed during Myc depletion (33), but BET inhibitors also disrupt cohesion-dependent DNA loops to activate lytic reactivation (34). We observe distinct effects of BET inhibitors on EBV transcription.

We propose a model (Fig. 1) wherein BET inhibitors control the EBV life cycle at two distinct points: before immediate-early gene expression and at initiation of lytic DNA replication. We suspect that the first block occurs indirectly because BET proteins do not themselves bind the *BZLF1* promoter (Fig. 3). JQ1 alters expression of genes controlled by the host protein BACH1, and BACH1 knockdown reduces viral reactivation (Fig. 2*F*). The second block likely occurs by directly preventing lytic DNA replication (Fig. 4), an association supported by binding of BRD2, BRD3, and BRD4 to the lytic origins of replication (Fig. 3). We favor the interpretation that JQ1 reduces BRD4 recruitment at the OriLyt elements to prevent replication initiation (Fig. 5), but we cannot formally rule out uncharacterized indirect effects. Although inhibition at the second block (Fig. 4*C*) is not as robust as inhibition at the first block (Fig. 2*A*), the combined effects result in complete suppression of all stages of the EBV lytic cycle (Fig. 2, *C* and *D*). Such dual-action inhibitors may be more effective than current drugs and result in a decreased risk of resistance. Many BET inhibitors are currently in clinical trials for cancer, and repurposing them against EBV may be useful in treating lytic replication during infectious mononucleosis and post-transplantation lymphoproliferative disorder.

Systems pharmacology approaches attempt to target key nodes in a network to most efficiently disrupt a biological process. The EBV lytic cycle progresses as a sequential cascade. Immediate-early expression of transcription factors to bind multiple viral promoters serves as an initial amplification step to activate early genes. DNA replication drives a second amplification step necessary for the transcription of late genes. Our studies of viral chromatin led to the serendipitous discovery that BET inhibitors target both key nodes in the EBV lytic cascade. These small molecules have several properties that may underlie the ability to perturb multiple steps in a viral pathway: nanomolar affinity for different complexes, involvement in epigenetics that dysregulates diverse genes, and binding to a host rather than a viral protein. Only a few other similar lead compounds also exist. GNF-2 targets both the Abl kinase and viral glycoprotein E to inhibit dengue virus replication (35). 17-Dimethylaminoethylamino-17-demethoxygeldanamycin reduces EBV replication in both a BGLF4-dependent and BGLF4-independent manner (36). Identifying even more compounds with multiple modes of inhibition would greatly increase our repertoire for treatment. We hope that BET inhibitors will serve as another prototype success to encourage directed polypharmacological discovery of next generation drugs.

## Experimental procedures

### Cell culture and treatment

MutuI (37) cells were grown under standard conditions (24). Akata-Zta (28) cells were obtained from Alison Sinclair (University of Sussex) and maintained in RPMI 1640 medium with 25 mm HEPES and 2 g/liter NaHCO_3_ supplemented with 10% (v/v) Tet system-approved fetal bovine serum (Clontech) in 5% CO_2_ at 37 °C. Growth was measured with a hemocytometer, and viability was measured by trypan blue exclusion.

To induce reactivation of the lytic cycle, log-phase cultures were treated with 10 μg/ml goat anti-human IgG, IgM, IgA secondary antibody (Thermo Fisher Scientific), 500 ng/ml doxycycline (Sigma-Aldrich), or vehicle for 1 day. Where BET inhibitor pretreatment is noted, 1 μm JQ1 (EMD Millipore), I-BET/GSK525762A (EMD Millipore), RVX-208 (Cayman Chemical), or vehicle was added for 1 h before induction. Where lytic genome replication elongation is specifically prevented, 200 μm acyclovir (Sigma-Aldrich) was added for 1 h before induction. The JQ1 dose response was determined by performing pretreatment with vehicle, 10 nm, 100 nm, 1 μm, or 10 μm for 1 h before induction with 1 μg/ml gemcitabine (Sigma-Aldrich) or vehicle for 3 days. Data were fit to the sigmoidal equation *a* + (*b* − *a*)/(1 + (*x*/*c*)*^d^*) (where *c* = IC_50_) using KaleidaGraph version 4.5.2.

Lentivirus shRNA was used to deplete BACH1 from MutuI cells. pLKO.1-hPGK-Puro-CMV-tGFP plasmids contained either a BACH1-targeting shRNA of the sequence CCGGCCAGCAAGAATGCCCAAGAAACTCGAGTTTCTTGGGCATTCTTGCTGGTTTTT (Sigma-Aldrich, TRCN0000013596) or a non-targeting control shRNA of the sequence CCGGGCGCGATAGCGCTAATAATTTCTCGAGAAATTATTAGCGCTATCGCGCTTTTT (Sigma-Aldrich, custom order). Lentiviruses were produced by transfecting plasmids into 293T cells, and infection-based titers were measured with GFP expression (38) (UCSF ViraCore). MutuI cells were transduced at a multiplicity of infection of 3 for 1 day, spun down, and resuspended in RPMI 1640 medium with 25 mm HEPES, 2 g/liter NaHCO_3_, 10% (v/v) fetal bovine serum, and 2 μg/μl puromycin in 5% CO_2_ at 37 °C.

To enrich for Akata-Zta cells containing latent or lytic EBV episomes, populations were separated based on cell surface marker expression. Reactivated cells were magnetically purified using MACSelect LNGFR MicroBeads and LS columns (Miltenyi Biotec).

### Staining and flow cytometry

EBV immediate-early gene expression was measured by flow cytometry on a FACSCalibur (BD Biosciences) after staining for BZLF1 (23).

### Western blotting

Protein knockdown was measured with Western blotting using the SuperSignal West Pico Chemiluminescent Substrate (Thermo Fisher Scientific), a ChemiDoc MP Imaging System (Bio-Rad), and ImageLab version 5.2.1 (Bio-Rad). BACH1 was detected using the F-9 antibody (Santa Cruz Biotechnology, sc-271211) at 1:300 dilution and rabbit anti-mouse-HRP (Abcam, ab6728) at 1:5000 dilution. For normalization, actin was detected using anti-β-actin (Abcam, ab8227) at 1:10,000 dilution and rabbit anti-mouse-HRP (Abcam, ab6728) at 1:10,000 dilution.

### RNA-seq

RNA-seq libraries were prepared and sequenced as described (23). EBV transcriptome profiles were determined by mapping reads to the viral genome (23). Every experimental condition was measured with three independent biological replicates and yielded ∼10–110 million mapped sequences per sample.

Differential expression of viral genes upon drug treatment was calculated by comparing independent triplicate experiments where Akata-Zta cells were induced with 500 ng/ml doxycycline for 1 day following 1-h pretreatment with either 1 μm JQ1, 1 μm I-BET, or vehicle. Transcriptional changes were calculated by measuring normalized nucleotide counts for each lytic transcript integrated over only exons spanning regions that did not overlap with any other transcript (43). The significance threshold was set at a *p* value <0.05.

Differential expression of human genes (39) upon drug treatment was calculated by comparing independent triplicate experiments where MutuI cells were treated with 1 μm JQ1 or vehicle for 1 day. Adaptors and low-quality portions of reads were trimmed with Fastq-mcf, sequence quality control was assessed with FastQC and RSeQC, and spliced and unspliced reads were aligned to the hg19 reference human genome with TopHat 2.0.13 and Bowtie 2.2.4, respectively. Reads were assigned to genes as annotated by Ensembl using featureCounts. Genes yielding counts per million expression below 0.5 or above 5000 in more than one sample were excluded from analysis. Renormalization of all other genes and calculation of differential expression *p* values were performed with edgeR. Pathway enrichment analysis was performed with GO-Elite 1.2.5 and a cutoff *p* value of 0.05. Transcription factors predicted to control enriched groups of genes were identified using a Z-score threshold of 2 from the MergedTFTargets gene set.

### ChIP-seq

ChIP-seq methods were based on standard protocols (40) with the following modifications. 3.6 × 10^7^ treated cells were cross-linked by adding formaldehyde to 1% (v/v) at room temperature for 10 min. Cells were then washed and lysed, and the resulting nuclei were frozen. Upon thawing, volumes were adjusted to 36% to reflect the starting cell number. Chromatin was digested with micrococcal nuclease for 5 min prior to shearing using a Bioruptor water bath sonicator (Diagenode). After sonication, tubes were vortexed and spun down for 10 min at 4 °C at 18,000 × *g*. Aliquots of input samples with 10 mm Tris, 1 mm EDTA, pH 7.5, added to 80 μl were combined with 100 μl of 50 mm Tris-HCl, pH 7.5, 10 mm EDTA, 1% (w/v) SDS and 20 μl of 20 mg/ml Pronase in 100 mm Tris, 150 mm NaCl, pH 7.5, for reverse cross-linking. 10–15 μl of epitope-specific antibodies per ChIP were preincubated with 150 μl of protein G Dynabeads (Invitrogen) and 450 μl of Buffer A containing 10 mm Tris-HCl, 1 mm EDTA, 150 mm NaCl, 5% (v/v) glycerol, 0.1% (w/v) sodium deoxycholate, 0.1% (w/v) SDS, 1% (v/v) Triton X-100, pH 8.0, rotating at 4 °C for 1 h. ChIP antibodies used recognized BRD4 (Bethyl Laboratories, A301-985A), BRD2 (Cell Signaling Technology, 5848S), BRD3 (Bethyl Laboratories, A302-368A), H3K27ac (Abcam, ab4729), H3K9ac (Abcam, ab4441), and H3K4me3 (Abcam, ab8580). Dynabeads were transferred to 2.5 ml of cold Buffer A without SDS but supplemented with 25 μl of Halt protease inhibitor mixture (Thermo Fisher Scientific). 277.5 μl of chromatin, the proportion equivalent to 1.5 × 10^7^ cells, was added to the beads in buffer and incubated on a rotator for 4 h at 4 °C. Beads were collected on a magnetic rack and washed consecutively with the following ice-cold buffers: Buffer A with Halt protease inhibitor mixture, then Buffer A with 500 mm NaCl and Halt protease inhibitor mixture, and finally 20 mm Tris-HCl, 1 mm EDTA, 250 mm LiCl, 0.5% (v/v) Nonidet P-40, 0.5% (w/v) sodium deoxycholate, Halt protease inhibitor mixture, pH 8.0. Complexes were eluted from the beads in 300 μl of 10 mm Tris, 1 mm EDTA, 0.7% (w/v) SDS, pH 8.0, at room temperature. For reverse cross-linking, the eluate was added to 450 μl of 50 mm Tris, 10 mm EDTA, 0.45% (w/v) SDS, pH 7.0, and 82.5 μl of 20 mg/ml Pronase in 100 mm Tris, 150 mm NaCl, pH 7.5. Reverse cross-linking of input and ChIP eluate samples was performed by incubation at 42 °C for 2 h and then at 65 °C overnight. DNA was purified by silica-based membrane affinity with a MinElute PCR purification kit (Qiagen).

ChIP-seq libraries were prepared with either the Ovation Ultralow Library System (NuGen) or Ovation Ultralow Library System V2 (NuGen). Quantification and size distribution of libraries were observed using the Bioanalyzer High Sensitivity DNA kit (Agilent). Size selection of the predominant DNA peak between ∼200 and 400 bp was performed either by gel extraction or magnetic bead purification. For gel extraction, the band from a 1% agarose gel in Tris acetate-EDTA was excised and purified with silica-based membrane affinity using a MinElute gel extraction kit (Qiagen). For magnetic bead purification, solutions were treated with two steps of solid-phase reversible immobilization using Agencourt RNAClean XP beads (Nugen). Libraries were sequenced on a HiSeq system (Illumina).

For immunoprecipitation experiments measuring the effect of JQ1 treatment on BRD4 occupancy in the MutuI line, 1 × 10^8^ treated cells were processed per ChIP. Initial buffer volumes were scaled up accordingly, but purification occurred with 10 μg of BRD4-specific antibody preincubated with 100 μl of protein G Dynabeads. Other steps were identical to the protocol performed with fewer cells.

Sequence fragments were trimmed to 50 bp and mapped to an index containing both the human hg19 and the EBV reference (GenBank^TM^ accession number NC_007605.1) genomes using Bowtie 0.12.8 (41) allowing for up to two mismatches and two alignments. Peaks on the EBV genome in MutuI cells were visualized after normalization to the background baseline (42). Peaks on the human genome in MutuI cells and EBV genome in Akata-Zta cells were visualized after normalization to the total number of mapped reads. We processed data from Akata-Zta cells treated with doxycycline differently to control for the emergence of lytic linear genomes that may confound determination of the background baseline representing circular episomes. Every experimental condition was measured with two independent biological replicates and yielded ∼20–80 million mapped sequences for each data set.

### EBV DNA quantitation

EBV genome abundance was determined by deep sequencing of total chromatin. Input chromatin was purified, libraries were prepared, and DNA was sequenced as described for ChIP-seq. The percentage of EBV reads was calculated as a proportion of viral reads that mapped to an index containing both the human hg19 and the EBV reference (GenBank accession number NC_007605.1) genomes using Bowtie 0.12.8 (41) allowing for up to two mismatches and one alignment. EBV genome abundance upon lytic induction and/or drug pretreatment was normalized to the vehicle control in each individual set of experiments. Every experimental condition was measured with three independent biological replicates and yielded ∼10–30 million mapped sequences for each data set.

### Replication fragment mapping

DNA content distribution across the EBV genome upon induction of lytic replication was measured by deep sequencing of total DNA. Total DNA was purified by silica-based membrane affinity as packaged in the DNeasy Blood and Tissue kit (Qiagen) and subsequently sheared using an S2 Focused ultrasonicator (Covaris) to obtain fragments ∼200 bp in length. Libraries were prepared, DNA was sequenced, and reads were mapped as described for ChIP-seq. Peaks on the EBV genome were visualized after normalization to the total number of mapped reads. Every experimental condition was measured with two independent biological replicates and yielded ∼30-40 million mapped sequences for each data set.

## Author contributions

K. M. K. and J. L. M. designed research. K. M. K., S. A. M., A. H., S. G. F., J. J. S., S. M. L., D. M. M., and J. L. M. performed research. K. M. K., S. A. M., A. H., S. G. F., S. M. L., D. M. M., and J. L. M. analyzed data. K. M. K. and J. L. M. wrote the paper.

## Footnotes

5 The abbreviations used are:

EBV: Epstein-Barr virus
BET: bromodomain and extraterminal
seq: sequencing
OriLyt: lytic origin of replication
H3K: histone H3 Lys
LNGFR: low-affinity nerve growth factor receptor.

## Note added in proof

In the version of this article that was published as a Paper in Press on June 6, 2017, an incorrect antibody was used to detect BACH1 expression in Fig. 2*E*. These experiments were subsequently repeated using an antibody against BACH1, and the figure has been corrected. This error does not affect the results or conclusions of this work.
